# My lifelong dedication to bile acid research

**DOI:** 10.1016/j.jbc.2023.104672

**Published:** 2023-04-03

**Authors:** John Y.L. Chiang

**Affiliations:** Department of Integrative Medical Sciences, Northeast Ohio Medical University, Rootstown, Ohio, USA

**Keywords:** bile acid synthesis, cholesterol 7α-hydroxylase, CYP7A1, FXR, TGR5, liver diseases

## Abstract

It is a great honor to be invited to write a reflections article on my scientific journey and lifelong bile acid research for the *Journal of Biological Chemistry*, in which I am proud to have published 24 articles. I have also published 21 articles in the *Journal of Lipid Research*, another journal of the American Society of Biochemistry and Molecular Biology. I begin my reflections from my early education in Taiwan, my coming to America for graduate study, and continue with my postdoctoral training in cytochrome P450 research, and my lifelong bile acid research career at Northeast Ohio Medical University. I have witnessed and helped in the transformation of this rural not so visible medical school to a well-funded leader in liver research. Writing this reflections article on my long and rewarding journey in bile acid research brings back many good memories. I am proud of my scientific contributions and attribute my academic success to hard work, perseverance, good mentoring, and networking. I hope these reflections of my academic career would help inspire young investigators to pursue an academic career in biochemistry and metabolic diseases.

I started my academic career in 1978 at the Northeastern Ohio Universities College of Medicine (NEOUCOM) in Rootstown, Ohio, a small rural town about 50 miles southeast of Cleveland. The school’s name was changed to Northeast Ohio Medical University, NEOMED, in 2011. I spent my entire academic career at NEOMED. Shortly after my official retirement from NEOMED in the summer of 2022, I was pleasantly surprised and honored to receive the invitation from Dr Patrick Sung on behalf of the *Journal of Biological Chemistry* (*JBC*) Classics and Reflections Committee to write this article, for which I was nominated by several senior colleagues. The following is the story of my lifelong academic journey and research focus on bile acid metabolism.

## Early education in Taiwan

I was born shortly after the Sino-Japanese war in 1947 in Nanjing, China. When I was 2 years old, my parents brought my older sister, one of my younger twin brothers, and me from mainland China to Taipei, a northern city in Taiwan. We were separated from my younger brother who was left behind with my uncle in China, almost 50 years until 1997 when my father became critically ill, thus allowing us to obtain a special permit for him to visit Taiwan. My brothers are identical twins raised under very different environments, yet they had the same height and same appearance and even the same habits after being separated for 50 years. My younger brother in Suzhou, China did not go to college during the cultural revolution and spent 3 years doing hard labor in the countryside in China. Both my parents went to radio technique school and worked for a news agency their entire life. They lived during the war time by moving from Nanjing to Kunming and then Chongqing to escape Japanese bombings. The war separated many families forever!

We settled in Taipei (means Northern City of Taiwan),Taiwan. My parents worked day and night shifts to raise three young children in difficult times. We lived in a small bedroom in a multifamily house and shared a bathroom and kitchen with three other families until I went to college. Our parents pushed us to study hard. At that time, we had only 6 years of mandatory education, equivalent to the completion of elementary school. Every student had to take a highly competitive entrance exam to be admitted to middle school, high school, and college, with only a small fraction of students moving onto the next level. I did not do well in the entrance exam to middle school and was admitted to the night class of Taipei’s Cheng-Kung Middle School. However, I excelled there and was offered direct admission to Cheng-Kung High School, a particularly prestigious institution in Taipei, without taking the entrance examination. Many teachers at this high school were college professors who taught me science and mathematics effectively. In my senior year of high school, my English teacher, Mrs Lee, had just returned from study in the United States. She arranged for our class to meet with an American military family in Taipei to experience American family life for a day. Although Mrs Lee was not a scientist, she told us that biochemistry was a hot field in the United States, and this had made a strong impression on me. In the 1950s to 1960s, Watson and Crick had discovered the structure of the DNA double helix, and the genetic code was soon deciphered thereafter. I was fascinated by these new discoveries and decided to study biochemistry in college and graduate school.

My class ranking in high school waived me from taking the entrance exam to enter Taiwan Chung-Hsing University in Taichung in 1965. Taichung, a city on the west coast of Taiwan, literally means “middle of Taiwan.” This provincial university (later became one of four national universities) was best known for its agricultural college. This was the first time I left home for a city new to me. I chose to major in Food Sciences in the Department of Agricultural Chemistry, which offered a distinctively “biochemical” curriculum including biochemistry, organic chemistry, analytical chemistry, microbiology, and nutrition, which were all interesting to me and helped prepare me well for my bile acid research later. I remember that an American Catholic priest who was the visiting professor from Georgetown University in Washington DC taught us special topics in biochemistry using the same English textbook, “Biological Chemistry” by Mahler and Cordes, that I would later use in my graduate studies in the United States. My college English teacher, Mrs Chi, had spent some time in the United States as a Fulbright Scholar in the literature at the University of Michigan. She encouraged students to go to the United States for graduate school, as at that time no university in Taiwan had a PhD program.

After graduation in 1969, I passed the National Examination for Studying Abroad and applied to graduate schools in the United States. The excellent education I received in Taiwan helped build a solid foundation for my graduate studies in the United States. My interest in biochemistry and my coming to the United States for graduate school were heavily influenced by my English teachers. Thus, the first lesson I learned, **L1: never underestimate the influence of your teachers, a principle that guided my own career as an academician and a mentor**.

## Coming to America for graduate school

**Figure 1:**
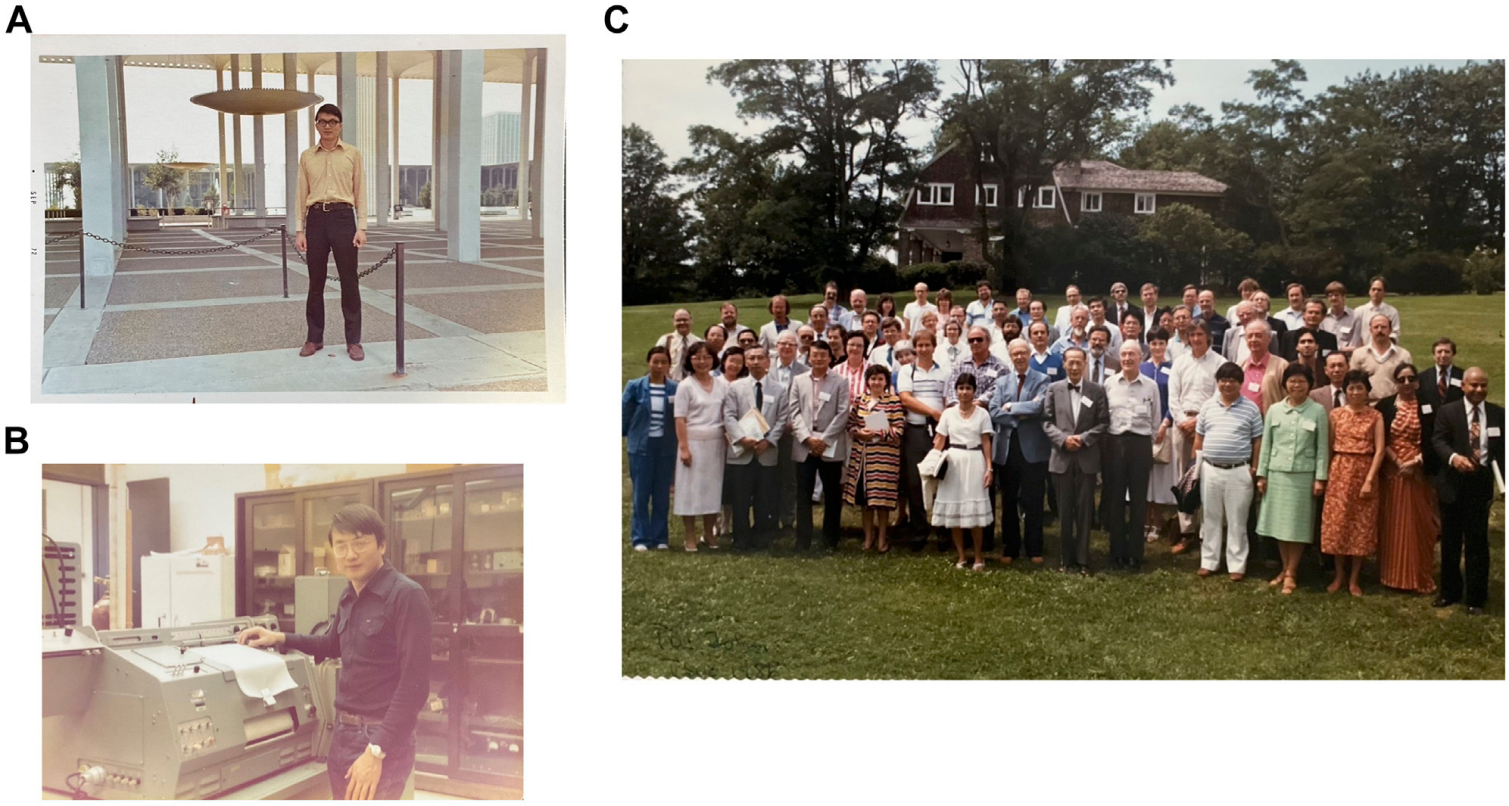
**Graduate student at the State University of New York (SUNY) at Albany.***A*, the author at the Academic Podium of SUNY at Albany, 1971. *B*, the author runs cytochrome spectra with Cary 17 spectrophotometer in 1972. *C*, group photo of the symposium in honor of Professor Tsoo E. King on the 30th Anniversary of the Reconstitution of the Respiratory Chain System. Tsoo E. King, center in bowtie; to his *left*, Britton Chance; the author, the *first row*, fourth from the *right*; Peter Mitchell, behind the author to the right. August 1 to 3, 1987, Rensselareville Institute, NY.

I received a tuition scholarship from the Department of Chemistry, State University of New York (SUNY) at Albany to help support my graduate studies. In 1970, after my completion of the 1-year mandatory military service in Taiwan, I took the first flight of my life to Albany, New York. At that time, the North Campus of SUNY at Albany was very modern and new (Fig. 1*A*). I lived in the international student dormitory, where I met students from different countries and was exposed to different cultures. I remembered that the next-door Taiwanese student who came to Albany with me had severe culture shock and went back to Taiwan after only one semester. However, I quickly adjusted to American life without much difficulty.

Although I went to a good school in Taiwan, the level of college education in Taiwan was not as advanced at that time, and the majority of college professors did not have a doctoral degree. As a result, my first year of study in Albany was not easy. The advanced organic chemistry, physical chemistry, and inorganic chemistry classes were highly comprehensive, and I had difficulty following the lectures. However, I studied hard and managed to pass my courses and PhD-qualifying exam in less than 2 years. After the first semester, Professor Tsoo E. King offered me a research assistantship to join his laboratory (Fig. 1*B*). Professor King had relocated his laboratory from Oregon State University 3 years earlier to serve as the Chair of the Department of Chemistry at SUNY at Albany. Many fellow graduate students warned me that Dr King was very strict and demanding; all his students took at least 6 years to complete their PhD. I remembered that Professor King required his graduate students to publish at least an article in the *JBC*, the premier journal of biochemistry at that time. Professor King was well known in the field of mitochondrial bioenergetics, and during my tenure in his laboratory, I had opportunities to meet many distinguished investigators in the mitochondria field, including Peter Mitchell, the 1978 Nobel Prize winner for Chemistry, and Britton Chance, an Olympic gold medalist in sailing, who designed many of the then-standard instruments in biochemistry, including the double beam spectrophotometer and stopped-flow apparatus, and many of the Who’s Who in bioenergetics shown in Figure 1*C*.

Professor King was trained in the laboratory of Professor David Keilin at Cambridge University. Keilin was an entomologist who discovered mitochondrial cytochromes in insect muscles using an optical prism–generated spectrum under a light microscope. In the King laboratory, I was assigned a project to purify cytochrome *c*1 in complex II of the mitochondrial respiratory chain. Professor King traveled a lot and did not have much direct contact with his students. Instead, he assigned me to work with two postdocs, Chang-An and Linda Yu. This couple patiently taught me all the research techniques I needed to succeed. They were hardworking and productive, and they were excellent mentors and my role models. From them, I learned **L2: my experience of being mentored by postdocs greatly influenced the training regimen I used for my own graduate students later**.

The biochemical approach to solve a complex system like the respiratory chain was to separate the electron transport enzymes and coenzymes to several complexes and then reconstituted the respiratory chain activity from isolated complexes. I went to slaughterhouses to collect beef hearts for preparing Keilin–Hartree bovine heart muscle submitochondrial particles to isolate cytochrome *b*–*c*1 complexes from hundreds of other proteins and macromolecules including additional cytochrome isoforms, nonheme iron proteins, and iron and sulfur proteins, as well as mitochondria-specific lipids, cardiolipin, ubiquinone, and coenzyme Q. I started empirically, performing ammonium sulfate fractionations, running huge columns packed with chromatographic matrices, conducting spectrophotometry to monitor the cytochromes in column fractions, and carrying out SDS-gel (using hand-packed glass tubes at that time) electrophoresis analysis to check the purity of proteins. I remembered that I flew with Professor King to New York City and then took a helicopter ride to the famous Bell Laboratory in Murrey Hill, New Jersey, to do an electron paramagnetic resonance spectroscopy (EPR) experiment with Jack Peisach to identify the heme iron ligand of cytochrome *c*1. I was impressed by the massive EPR instrument, which filled the entire room with a huge magnet. There were wires and vacuum tubes everywhere. To my disappointment, the EPR spectrum showed a strong signal of inorganic copper instead of heme iron in cytochrome *c*1. Nevertheless, it was a memorable trip and a very cool experience riding in a helicopter over the Manhattan skyscrapers.

I worked hard as a graduate student, 6 days a week and some evenings in the laboratory. I remembered giving my first scientific presentation at the Federation of American Societies of Experimental Biology meeting in 1974 in Atlantic City, New Jersey. At that time, poster presentation had not been utilized, and all presentations were oral. That was the first time I spoke on the podium, but I was not intimidated, and my presentation did not disappoint Professor King. He told me I did a good job, quite a compliment from a strict mentor. In the end, I published four articles in the *JBC* and two other articles with Professor King. These articles report the purification and properties of mammalian cardiac cytochrome *c*1 and its interaction with cytochrome *c*. I met my wife Lisa in New York City, and we got married in 1973. I graduated in 1976, the first and only PhD recipient in my family, which makes me proud. Looking back, I received excellent graduate training at SUNY at Albany under the tutelage of a well-known and well-connected investigator in the field. SUNY at Albany may not be a flashy school, but I learned **L3: less well-known universities are perfectly capable of providing excellent education and research experiences**.

## My postdoctoral training in cytochrome P450

After passing my PhD defense, I asked Professor King “Where should I go for my postdoctoral training?” He advised me to work with Professor Minor Jud Coon at the Department of Biological Chemistry at the University of Michigan in Ann Arbor, Michigan. After a phone call between Professors King and Coon, and without an interview, Dr Coon called to offer me a postdoctoral scholar position in his laboratory. I rented a small U-Haul trailer and drove with my pregnant wife to Ann Arbor to start my postdoctoral training in July 1976. The University of Michigan is a much bigger university compared with SUNY at Albany. Professor Coon’s laboratory was the first to demonstrate that cytochrome P450s (CYPs) are involved in the oxidation of endogenous substrates such as fatty acids, in addition to drugs and carcinogens. Anthony Lu in Jud’s laboratory had isolated CYP-reductase, P450s, and phospholipid fractions from rabbit liver microsomes to reconstitute a lauric acid ω-hydroxylation system (also published in the *JBC*). The microsomal monooxygenase system seemed to be much simpler compared with mitochondrial oxidative phosphorylation machinery involving hundreds of enzymes and proteins. However, multiple CYP isoforms were isolated from the liver and other organs/tissues, and more than 100 P450 enzymes with overlapping substrate specificities have been identified in liver and other tissues of different species by many investigators.

**Figure 2:**
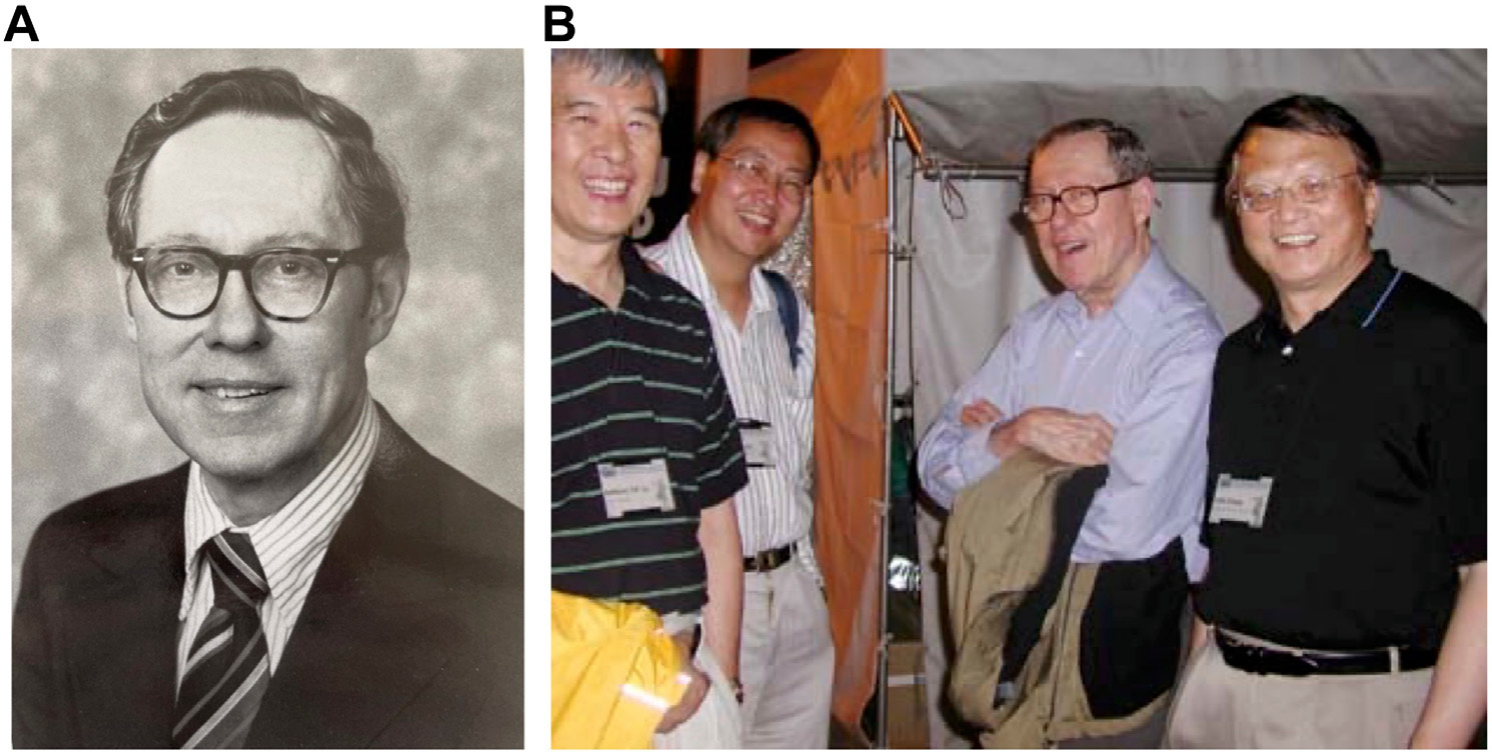
**My postdoc mentor Professor Minor J. Coon.***A*, Professor M. J. Coon. *B*. Dr. Coon at Microsome and Drug Oxidation Meeting in Sapporo, Japan, 2002. Anthony Lu is to his left, author is to his right.

Jud Coon was the chairman of the Department of Biological Chemistry at the time (Fig. 2*A*) and had senior technicians to train new postdocs and graduate students. He tasked a senior postdoc, Ron White, to manage an efficient and a productive laboratory. I applied for and received an individual National Research Service Award from the National Institutes of Health (NIH) to support my research in the Coon laboratory. There I learned how to isolate microsomes and purify rabbit liver CYPs, and I studied the enzyme kinetics and mechanisms of P450 in drug metabolism. Jud managed his laboratory very differently from Professor King. We had regular laboratory meetings to discuss research projects, and we celebrated the birthday of each laboratory member with ice cream and cake and had annual summer picnic and year-end party. Jud served as the president of American Society for Biochemistry and Molecular Biology and the International Union of Biochemistry and was elected to the US National Academy of Sciences in 1983. His scientific achievements and leadership had great influence on all his trainees, including Anthony Lu (Merck, Co) (Fig. 2*B*), Paul Hollenberg (University of Michigan), F. Peter Guengerich (Vanderbilt University), Eddy Morgan (Emory University), and Xing-Xing Ding (University of Arizona), among others. They became the leading investigators in the P450 field. I attended many international microsome and drug oxidation meetings and meetings of International Society for Study of Xenobiotics, where I met many distinguished P450 investigators, who had great influence on my research in drug metabolism. I learned how to write articles from Jud; he patiently went sentence by sentence in my draft articles with me. Jud loves music and arts. When he came to Cleveland, he went to Cleveland Museum of Arts to see the Chinese painting collections. NEOUCOM presented Dr Coon an honorary doctoral degree in 1980 for his contribution to P450 research program at NEOUCOM. Jud passed away in 2018 at the age of 97. He was a great mentor and an influential leader in P450 field. I learned **L4: it is important to pursue postdoctoral training under the mentorship of an influential leader in the field and to emulate that mentor’s management style to run an efficient and productive laboratory**.

## Starting my academic career at Northeast Ohio Medical University

In early 1978, Jud received a letter from Dr Ferenc Hutterer, the Program Chief of Biochemistry and Molecular Pathology at NEOUCOM in Rootstown, Ohio, to seek nominations for an assistant professor position. This job specifically required a candidate with experience in the purification and enzymology of CYP. Several postdocs senior to me had no interest in applying for a faculty position at a new and relatively small medical college in rural Ohio, and so they passed this job opportunity to me. Around that time, the United States was experiencing double digit inflation and high unemployment. It was difficult for a foreign scientist to find a faculty position. I thought, why not drive 3 h to visit NEOUCOM? At the time, NEOUCOM was a community-based medical school with an accelerated BS/MD program in consortium with three Northeast Ohio Universities: Kent State University, the University of Akron, and Youngstown State University. NEOUCOM was established in 1973, and the Rootstown campus opened in 1976. I was offered the job on the spot. I accepted the job offer. In August 1978, I rented a small U-Haul truck (this time we had some furniture) and drove with my wife and baby boy to Ohio to start my academic career at NEOUCOM. Most faculty were recruited from famous institutes such as Mount Sinai Medical College, Baylor College of Medicine, and the NIH. At that time, NEOUCOM was not diverse, faculty, administrators, and medical students were predominantly white Caucasian men. I was the second Asian faculty hired at NEOUCOM, and, at the time, never thought I would spend my entire academic career there.

As a newly established community-based medical school, it was not surprising that the primary duty for faculty at NEOUCOM was teaching. The Program in Biochemistry and Molecular Pathology at NEOUCOM involved teaching medical biochemistry to a class of 45 medical students who had finished 2 years of phase 1 undergraduate courses and advanced to the second phase of medical education. We used an organ-based approach to teach intermediate metabolism: for example, the liver for metabolic pathways, the red blood cell for protein structure and function, the muscle for energy metabolism, and so on. We had weekly case studies, which were designed for students to learn problem solving by applying basic biochemical knowledge for analysis of clinical laboratory data and diagnosis of metabolic diseases, pathogenic mechanisms, and potential treatments. I spent about 20 h a week teaching, which was a heavy load for an assistant professor.

At NEOUCOM, I also had the opportunity for research as long as I fulfilled my teaching responsibilities. My focus on cholesterol and bile acid metabolism was inspired by Ferenc Hutterer, MD, the Director of the teaching Program of Biochemistry and Molecular Pathology. During World War II, Ferenc Hutterer and family had escaped from a concentration camp in Poland to Vienna. Later he became a medical student at the University of Szeged, Hungary, worked in the laboratory of Nobel Laureate and biochemist Albert Szent-Gyorgi. The Hungarian Revolution in 1956 forced the Hutterer family to leave Hungary to New York City. He joined Hans Popper’s Department of Pathology at Mount Sinai Hospital in New York as the director of the chemistry laboratory. Hans Popper is generally regarded as the founding father of hepatology. Ferenc Hutterer gained research experience in bile acid analysis when he was in Hans Popper’s laboratory, and he wanted to establish a focused research program to study cholesterol and bile acid metabolism at NEOUCOM. Ferenc’s idea was to have an enzymologist to purify cholesterol 7α-hydroxylase (CYP7A1), a molecular biologist to clone Cyp7a1 complementary DNA (cDNA), a biochemist to study P450s in drug metabolism, and an organic chemist to study cholesterol in membrane structure and function. Before I joined the faculty, Ferenc Hutterer and four other investigators submitted a program project application on bile acid metabolism to NIH. The project was not funded. While I thought it might be premature for a new medical school investigator to apply for a program project grant, it showed Hutterer’s ambition and vision for bile acid research at NEOUCOM. Dr Hutterer retired from NEOMED in 1997 after a long and decorated career. He passed away in 2017. In honor of Dr Hutterer’s contribution to liver research at NEOMED, I raised funds and organized The Ferenc Hutterer, MD Seminar Series in Liver Research in 2018. Dr Frank Gonzalez from National Cancer Institute was the first speaker in this seminar series.

## Starting CYP and bile acid research

When I arrived at the Rootstown campus, the laboratories were just completed and equipped with everything I needed for my research. I remembered having to write a research proposal to get $7000 from the biomedical research support grant (which was abolished a few years later) from NIH to NEOUCOM. This was a very small startup package compared with the startup packages for assistant professors now. Therefore, faculty had to collaborate with each other on focused research areas to make efficient use of shared equipment and limited resources. I spent half of my startup on the purchase of reagents and glassware. I applied for and received several small grants from the American Heart Association Ohio Valley Chapter and the United Way campaign as well as a starter grant from the Pharmaceutical Manufacturers Association Foundation. These small grants allowed me to hire a part-time technician to start my research projects on CYP in drug metabolism and atherosclerosis.

Rabbits were a popular animal model for these studies because of the ease of isolating CYPs from their large liver. Hamsters were used to study gallstone disease because of a similar bile acid composition to that in human bile and their susceptibility to cholesterol gallstones when fed a lithogenic diet. My first graduate student Thung-S. Lai (MacKay Medical College, New Taipei, Taiwan) started the purification and cloning of 3-methylcholanthrene-induced hamster P450s and studied their metabolism of aflatoxin B1 (1, 2). We also partially purified a P450 from cholestyramine-treated rabbit and hamster livers and carried out reconstitution with NADPH–P450 reductase to show that it catalyzes the 7α-hydroxylation of cholesterol (3). The preliminary data generated from these endeavors were used in an NIH R01 grant application to study the regulation of hamster P450. The grant was funded in 1983. The grant had a small budget of about $60,000 yearly direct costs, for 3 years. Funding of this NIH grant allowed me to hire my first postdoc and a fulltime technician and provided a sufficient budget to begin cloning CYP7A1 cDNA.

## Bile acid synthesis and metabolism in the gut to liver axis

**Figure 3:**
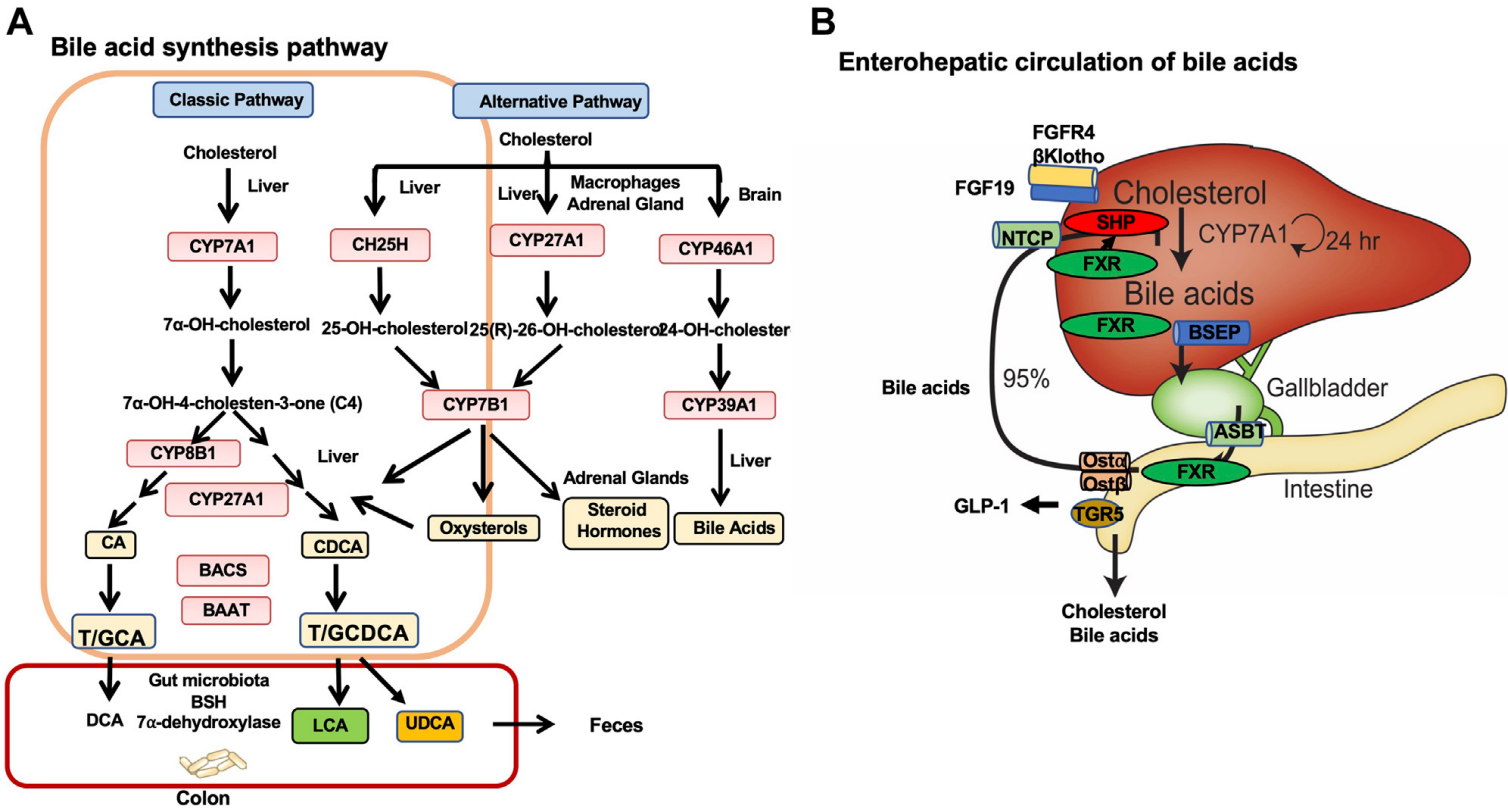
**Bile acid synthesis and regulation.***A*, bile acid synthesis pathways. Key regulatory enzymes in the classic pathway and alternative pathways are shown. Cholic acid (CA) and chenodeoxycholic acid (CDCA) are the two primary bile acids synthesized in the liver. They are conjugated to taurine (T) and glycine (G) by bile acid CoA synthase (BACS) and bile acid amino acid transferase (BAAT) and secreted into the gastrointestinal tract. In intestinal lumen, gut bacteria bile salt hydroxylase deconjugates bile acids, and bacteria 7α-dehydroxylase removes the hydroxy group from CA and CDCA to form the secondary bile acids, deoxycholic acid (DCA) and lithocholic acid (LCA), respectively. CDCA is epimerized to ursodeoxycholic acid (UDCA)*.B*, the proposed mechanisms of bile acid feedback inhibition of bile acid synthesis. In the liver, bile acids activate farnesoid X receptor (FXR) to induce SHP, which inhibits CYP7A1 gene transcription. In the intestine, FXR induces fibroblast growth factor (FGF19 in humans or FGF15 in mice), which is transported to the liver to activate FGF receptor 4–βKlotho complex to inhibit CYP7A1 gene transcription. Bile salt export pump (BSEP), apical sodium-dependent bile salt transporter (ASBT), organic solute transporter α and β (OSTα/β), and sodium taurocholate cotransport peptide (NTCP) are shown.

Bile acids are natural detergent molecules derived from cholesterol in the liver through at least 17 enzymatic reactions occurring in microsomes, mitochondria, peroxisomes, and the cytosol. CYP7A1 is the first and rate-limiting enzyme in the classic bile acid biosynthesis pathways (Fig. 3*A*). After synthesis, the primary bile acids cholic acid (CA) and chenodeoxycholic acid (CDCA) are conjugated to taurine or glycine to form T/GCA and T/GCDCA, which are excreted into the bile canaliculi to form mixed micelles with cholesterol and phospholipids. Bile acids are stored in the gallbladder, and in the postprandial state, and are excreted into the gastrointestinal tract to facilitate the absorption of nutrients, lipids, and drugs. In the ileum, about 95% of bile acids are reabsorbed and transported *via* portal blood circulation to the liver to inhibit bile acid synthesis. In the colon, gut bacteria that express bile salt hydrolase activity deconjugate taurocholic acid (TCA) and taurochenodeoxycholic acid, then bacterial 7α-dehydroxylases covert them to deoxycholic acid (DCA) and lithocholic acid (LCA), respectively. In humans, CA, CDCA, and DCA are the major bile acids in the circulating bile acid pool, whereas in mice, the major bile acids are CAs and muricholic acids. The underlying molecular mechanism of the enterohepatic circulation of bile acids from the gut to the liver to inhibit CYP7A1 and bile acid synthesis is not entirely known. Transport of bile acids from the liver to the intestine and back to the liver involves specific bile acid efflux transporters and uptake transporters in the liver and ileum (Fig. 3*B*). It was imperative to isolate and purify cholesterol 7α-hydroxylase to study cholesterol and bile acid metabolism in health and diseases. This was the impetus of my early work of bile acid metabolism at NEOUCOM.

Several studies published in the 1960s to 1970s suggested that cholesterol was the substrate for cholesterol 7α-hydroxylase in the rat liver microsome, and that cholesterol 7α-hydroxylase is a monooxygenase that uses NADPH to provide one electron to reduce an oxygen atom to form a hydroxyl group stereospecifically at the 7α-position of the cholesterol steroid ring to 7α-hydroxycholesterol (4, 5). This enzyme activity is inhibited by CO binding to the ferrous iron of the heme group to exhibit a unique absorption peak at 450 nm, which is typical for CYP monooxygenases. Several animal studies in the 1950s demonstrated that biliary bile diversion, or feeding the bile acid sequestrant cholestyramine to rats, stimulated the low-density lipoprotein receptor–mediated uptake of serum cholesterol to the liver to induce cholesterol 7α-hydroxylase activity and bile acid synthesis to reduce serum cholesterol. Conversely, CA feeding inhibited bile acid synthesis by inhibiting cholesterol 7α-hydroxylase activity. Cholestyramine (Questran) was subsequently approved by the Food and Drug Administration in 1973 and prescribed as a drug to lower serum cholesterol in hypercholesterolemic patients with a high risk of heart and gallstone disease at that time. The study of cholesterol and bile acids in the pathogenesis of cholesterol gallstone disease and therapy was an emerging area of research when I started my laboratory at NEOMED.

## The race to purify and clone CYP7A1

I was at the forefront of the race to purify and clone CYP7A1 in the 1980s, which was one of the most exciting and rewarding experiences in my research career. I was promoted to full professor in 1988, but still working at the bench and personally involved in purifying CYP7A1 with my first technician Wynne Miller and the cloning of CYP7A1 cDNA with my second graduate student, Yan-Chun Li.

### Purification of CYP7A1 enzyme

Rats do not have gallbladders, but they do have a relatively long intestine, and they have a much larger (∼100 times greater) circulating bile acid pool and higher CYP7A1-specific activity compared with mice, rabbits, or hamsters. CYP7A1 is the rate-limiting enzyme in bile acid synthesis, thus its expression level tends to be very low, and its mRNA has a short half-life of about 2 to 3 h. For these reasons, we used rats for enzyme purification. The isotope labeling and thin-layer chromatography– and gas chromatography–based assays of CYP7A1 activity were tedious and not sensitive for this low-abundant enzyme. To facilitate our effort, I developed a simpler and more sensitive reversed-phase HPLC-based assay for cholesterol 7α-hydroxylase activity (6). Purification of CYP7A1 turned out to be very challenging. Because cholesterol 7α-hydroxylase activity exhibits a distinct diurnal rhythm, peaking in the middle of the dark cycle and reaching a nadir in mid-day, we reversed the light cycle (darkness from 9 AM to 9 PM) to sacrifice rats at 9 AM in the dark period. We fed animals a 5% cholestyramine-enriched diet to induce CYP7A1 for 2 weeks and isolated liver microsomes for protein purification. It took us about 3 years and 200 rats to finally purify CYP7A1 protein to a single band in SDS-gel electrophoresis. I could only obtain about 200 μg purified CYP7A1, which I used to raise polyclonal antibodies in rabbits. We were able to use these antibodies to demonstrate that cholestyramine-induced cholesterol 7α-hydroxylase activity paralleled CYP7A1 protein level. We published the purification of CYP7A1 and immunochemical evidence for its circadian rhythm and induction by cholestyramine in 1990 in the *JBC* (7), the first *JBC* article published by my own laboratory.

### Sabbatical leave to learn molecular biology

In addition to biochemically purifying CYP7A1, I also had the ambitious goal of cloning the CYP7A1 cDNA. In the 1970s to 1980s, molecular biology techniques were developed for cloning and sequencing of cDNAs and coding genes, and many drug-metabolizing CYP450 cDNAs were successfully cloned. I knew several groups had tried to clone CYP7A1 cDNA, but no one succeeded. My molecular biologist colleagues tried but failed to clone CYP7A1 cDNA using the degenerative nucleotide probes based on the available CYP450 N-terminal amino acid sequences, a common approach to clone members of a gene family. I thought it was necessary for me to learn and adopt molecular biology techniques to be competitive. When I was eligible for sabbatical leave in 1986, I left behind my wife and two young children and went to Cecil Pickett’s laboratory at Merck Research Laboratory in Rahway, New Jersey, for 6 months, to learn molecular biology and cloning techniques. In the Pickett laboratory, I learned how to isolate poly A-mRNA, construct cDNA plasmids, carry out restriction enzyme digestion, and screening of cDNA libraries.

### Cloning of CYP7A1 cDNA

Six months later, I returned to Rootstown to transform my laboratory to a ribonuclease-free molecular biology laboratory, and I also successfully renewed my NIH grant for three more years in 1987. I remembered vividly that when I was trying to recruit Yan Chun, I told him we were going to embark on a very difficult project to clone CYP7A1 cDNA but, if we succeeded, we would become famous. He said confidently to me “no problem, Dr Chiang, I can do it.”- With this spirit, YanChun and I isolated liver polysomes from cholestyramine-fed rats and enriched CYP7A1 mRNA in the polysomes with mono-specific antibodies against CYP7A1 that I had previously developed. Isolated poly A-mRNAs were reverse-transcribed to cDNA to construct an expression cDNA library. We screened the expression library with antibodies to pick up a few positive clones expressing the CYP7A1 protein. *In vitro*– transcription coupled to in vitro translation confirmed that every positive clone we obtained expressed CYP7A1 mRNA and protein. We poured urea sequencing gels for manual Sanger sequencing of CYP7A1 cDNA and reading autoradiographic films for DNA sequences by eye. I had a small laboratory, but it took us only 2 years to clone CYP7A1 cDNA. In October 1989, Professor Kyuichiro Okuda’s laboratory at the Hiroshima University published a human CYP7A1 cDNA sequence in FEBS Letter (8). We immediately submitted our article on the cloning, sequencing, and regulation of cholesterol 7α-hydroxylase to the *JBC* on November 10, 1989. The revised article was submitted on March 20, 1990, accepted in 2 weeks, and published in the July 15 issue of the *JBC* (9). At that time, an accepted article was not released until published in the printed form. Professor David Russell from the University of Texas Southwestern Medical School submitted their rat Cyp7a1 cloning article on January 9, 1990, and published in the May 15 issue of the *JBC*, just 2 months before us (10). Russell’s approach was to partially purify CYP7A1 from liver microsomes of rats fed cholestyramine or CA. A protein band induced by cholestyramine but repressed by CA on SDS gels was selected for the N-terminal amino acid sequencing to design degenerative nucleotide probes for screening rat liver cDNA libraries. David told me later that he used 2000 rats to purify and clone CYP7A1! Thus, three laboratories independently cloned the CYP7A1 gene almost simultaneously and confirmed each other’s CYP7A1 cDNA sequences. Cloning of CYP7A1 cDNA was considered a breakthrough in bile acid research in the 1990s. Both Okuda and Russell were awarded the prestigious Windaus Prize by the Falk Foundation of Germany for their contribution to cholesterol and bile acid research.

I am very proud of our accomplishment in the purification and cloning of CYP7A1 so quickly with little prior experience in cDNA cloning and the limited resources available to me. These experiences taught me that we must be prepared to learn new techniques to advance our research agenda. I recommend highly to all investigators to take advantage of sabbatical leave to update their knowledge base and learn new technologies. My success in cloning CYP7A1 cDNA also taught me **L5: an investigator with few resources can compete with highly experienced investigators with larger laboratories at the most prestigious research institutes**.

## Studying the complex mechanism of bile acid feedback regulation of bile acid synthesis

With the successful cloning of CYP7A1 cDNA, we wanted to study the molecular mechanism of bile acid regulation of *CYP7A1* gene expression. From the 1980s to 1990s, P450 was an emerging area of active research. Investigators focused on purification and cloning of CYPs to study the structure and mechanisms of P450 in drug metabolism. The high-resolution structure of soluble bacterial P450cam was solved in 1987. Many P450 investigators tried to solve the structure of mammalian P450s, which are membrane proteins and difficult to express in large quantity and solubilize. We successfully expressed a soluble form of rat CYP7A1 in *Escherichia coli* by deleting amino acid residues 2 to 24, which harbor the putative membrane binding and targeting signal. The truncated CYP7A1 was purified to a single band on SDS gel and was catalytically active when reconstituted with NADPH–cytochrome reductase. The study was published in the *JBC* in 1991 and considered a classic article in P450 research (11). A similar approach was used to express a truncated human CYP7A1 in *E. coli* (12). I was hoping to use the soluble CYP7A1 protein to solve the crystal structure of this enzyme. However, the expression levels and the heme content of soluble CYP7A1 were low and not suitable for crystallization. I decided to focus our research on understanding the mechanism of transcriptional regulation of the *CYP7A1* gene. Our effort led to the cloning of the rat, human, and hamster *CYP7A1* genes and analysis of gene structures including determination of the 5′-flanking gene promoter sequences (13, 14, 15).

### Transcriptional regulation of CYP7A1

The availability of CYP7A1 cDNA probes allowed us to study the transcriptional regulation of CYP7A1 mRNA by Northern blot hybridization in animals and in hepatocyte cultures. We collaborated with Professors Reno Vlahcevic and Phil Hylemon and a research fellow Michael Pandak, MD at the Medical College of Virginia (now Virginia Commonwealth University School of Medicine) in Richmond, VA and used a chronic bile fistula rat model to show that biliary diversion of bile acids increased CYP7A1-specific activity; intraduodenal infusion of taurocholate suppressed CYP7A1 mRNA expression, whereas cholesterol feeding increased CYP7A1-specific activity and mRNA levels (16). Further study showed that taurocholate inhibited CYP7A1 by downregulating the expression of β-hydroxy β-methylglutaryl-CoA reductase, the rate-limiting enzyme in *de novo* cholesterol synthesis, whereas infusion of mevalonate could overcome taurocholate inhibition of CYP7A1 expression in bile fistula rats (17).

### Bile acid response elements

We were the first laboratory to study the *CYP7A1* gene promoter in the 1990s. We first mapped the *CYP7A1* gene proximal promoter using DNase I footprinting assay to identify potential sequences for transcription factor binding. We found that these sequences contain several AGGTCA-like direct repeat (DR) motifs found in many genes responsive to steroid hormone stimulation (18). My postdoc Diane Stroup (Kent State University) developed gel retardation assays using nucleotide sequences containing a DR with four base spacings (DR4 motif) from rat and human *CYP7A1* gene promoters. We performed DNA mobility shift assays using nuclear extracts from rat liver and demonstrated that bile acid feeding reduced, whereas cholesterol and cholestyramine feeding increased the binding of the transcription factors to rat and human DR4 sequences. Another postdoc Maurizio Crestani (University of Milan, IT) and Diane Stroup developed *CYP7A1* gene promoter luciferase reporter assays and mutagenesis analysis to show that these two footprints harbor sequence elements that confer responsiveness to bile acid repression. We thus named these two footprints the bile acid responsive element I (BARE-I) and II (BARE-II) (19, 20, 21, 22). To our surprise, we found that human *CYP7A1* gene transcription was not regulated by hormones or the transcription factors we tested (23). Interestingly, the Mangelsdorf laboratory reported that CYP7A1 expression levels in the liver of *Lxr*-knockout mice were not induced and that LXR/RXR bound to a DR4 motif in the BARE-I of mouse *Cyp7a1 gene* promoter we identified (24, 25). We found, however, that LXR did not stimulate human *CYP7A1* promoter luciferase activity, because the human *CYP7A1* gene promoter lacks the DR4 motif (26). Our article was rejected by several journals, including the *JBC* and the *Journal of Lipid Research* (*JLR*). We instead published it in *Gene* (26). To date, this article has been cited 475 times and is my most cited original article that I have published. I learned from this experience **L6: while it is nice to publish in high-impact journals, an article in a less prestigious journal also can become highly cited**.

### Bile acid–activated farnesoid X receptor in regulation of CYP7A1

In 1999, farnesoid X receptor (FXR) was identified as a bile acid–activated receptor and bile acid sensor (27, 28). FXR is activated by primary bile acids and is highly expressed in the gastrointestinal tract as a master metabolic regulator of not only bile acid synthesis and transport but also lipogenesis, gluconeogenesis, and NF-κB-mediated inflammation in hepatocytes and macrophages. In 2000, Frank Gonzalez’s laboratory (National Cancer Institute) generated FXR knockout mice to demonstrate that FXR plays a critical role in bile acid homeostasis and lipoprotein metabolism (29). In 2000, we published an article in the *JBC* reporting that FXR inhibition of *CYP7A1* gene transcription required BARE-II, but FXR did not bind to this element in the *CYP7A1* gene promoter, and FXR inhibition of CYP7A1 must occur *via* an indirect mechanism (30). This *JBC* article has been cited over 350 times. Subsequently, a cascade mechanism was proposed, in which FXR induces the expression of the negative receptor, short heterodimer partner, to inhibit the transactivation of CYP7A1 by another nuclear receptor, liver-related homolog (31, 32) (Fig. 3*B*). In collaboration with Grace Guo (Rutgers University) and Frank Gonzalez using whole body and intestine-specific *Fxr* knockout mice, we demonstrated that the intestine FXR-induced fibroblast growth factor 15 (FGF15) to liver FGF receptor 4 pathway is critical for suppressing both CYP7A1 and sterol 12**α**-hydroxylase (CYP8B1) expression, whereas the liver FXR/short heterodimer partner pathway may be important for suppressing CYP8B1 expression but may play only a minor role in CYP7A1 expression. This *JLR* article has been cited 348 times.

### Intestinal factors in bile acid feedback regulation

Both obstruction of the common bile duct and biliary drainage induce CYP7A1 activity, suggesting the presence of bile acids in the intestine may be required for inhibition of bile acid synthesis. In 1995, Pandak *et al*. (33) reported that intravenous infusion of taurocholate to bile fistula rats failed to downregulate CYP7A1-specific activity, whereas intraduodenal infusion of taurocholate repressed CYP7A1 activity, suggesting that an intestine-secreted humoral factor might be required to mediate the negative feedback regulation of bile acid synthesis in the liver. However, this article published in *Gastroenterology* did not receive much attention from investigators in the field. Ten years later, FGF15 (FGF19 in humans) was identified as an enteroendocrine hormone that is induced by intestinal FXR to activate hepatic FGF receptor 4–βKlotho complex signaling to inhibit *CYP7A1* gene transcription (Fig. 3*B*) (34). FGF15 is a postprandial hormone expressed in mouse intestine but not in mouse liver. Interestingly, we found that in primary human hepatocytes, FGF19 could be induced by CDCA and GW4064, a potent FXR agonist, to inhibit CYP7A1 mRNA transcription (35). This study showed that FGF19 activates the mitogen-activated protein kinase/extracellular signal–regulated kinase 1/2 pathway to mediate FGF19 inhibition of *CYP7A1* gene transcription.

## CYP7A1 and bile acids in liver metabolism and diseases

In addition to the cloning and characterization of CYP7A1, my other interest and proud contribution to the field is the implications of CYP7A1 and bile acids in liver metabolism and diseases. Inborn errors of bile acid synthesis have been identified in 13 of the 17 enzymes involved in bile acid synthesis (see review in Ref. (36)). In neonates, mutations in and deficiency of bile acid synthesis enzymes cause malnutrition and accumulation of toxic steroid intermediates, which cause liver injury and cholestatic liver disease. To date, only one family of patients with a frame shift mutation in the *CYP7A1* gene has been reported (37). These patients have dyslipidemia, premature atherosclerosis, gallstone disease, and reduced CA and DCA, which are likely synthesized *via* the alternative bile acid synthesis pathway.

I recruited a highly talented graduate student, Tiangang Li, to my laboratory in 2001. Tiangang found that low physiological concentrations of insulin stimulate CYP7A1, but at high concentrations, insulin inhibits CYP7A1 mRNA expression *via* FOXO1 and SREBP-1c in human hepatocytes (38). FOXO1 also inhibits CYP7A1 expression in human primary hepatocytes and in high fat diet–fed mice (39). Glucose stimulates CYP7A1 expression in human hepatocytes by epigenetic mechanisms, by increasing histone acetylation, and by decreasing methylation of the *CYP7A1* chromatin (40). Tiangang (41) then studied the effect of nutrients on CYP7A1 expression and found that fasting reduces CYP7A1 expression, whereas refeeding rapidly induces CYP7A1 activity, protein, and mRNA expression levels. Refeeding stimulates AKT phosphorylation, involved in insulin signaling, and increases histone acetylation while reducing repressive histone methylation of *CYP7A1* chromatin. This study also showed that hyperglycemic streptozocin-induced type 1 diabetic mice, or genetically obese ob/ob mice had increased histone acetylation of *CYP7A1* chromatin, leading to increased *CYP7A1* expression and bile acid pool size. These studies suggest that glucose and insulin are major postprandial factors that induce *CYP7A1* expression and bile acid synthesis by epigenetic mechanisms. Tiangang published 10 articles in my laboratory and was recruited to University of Kansas as an assistant professor in 2012.

### Bile acid synthesis in liver disease models

**Figure 4:**
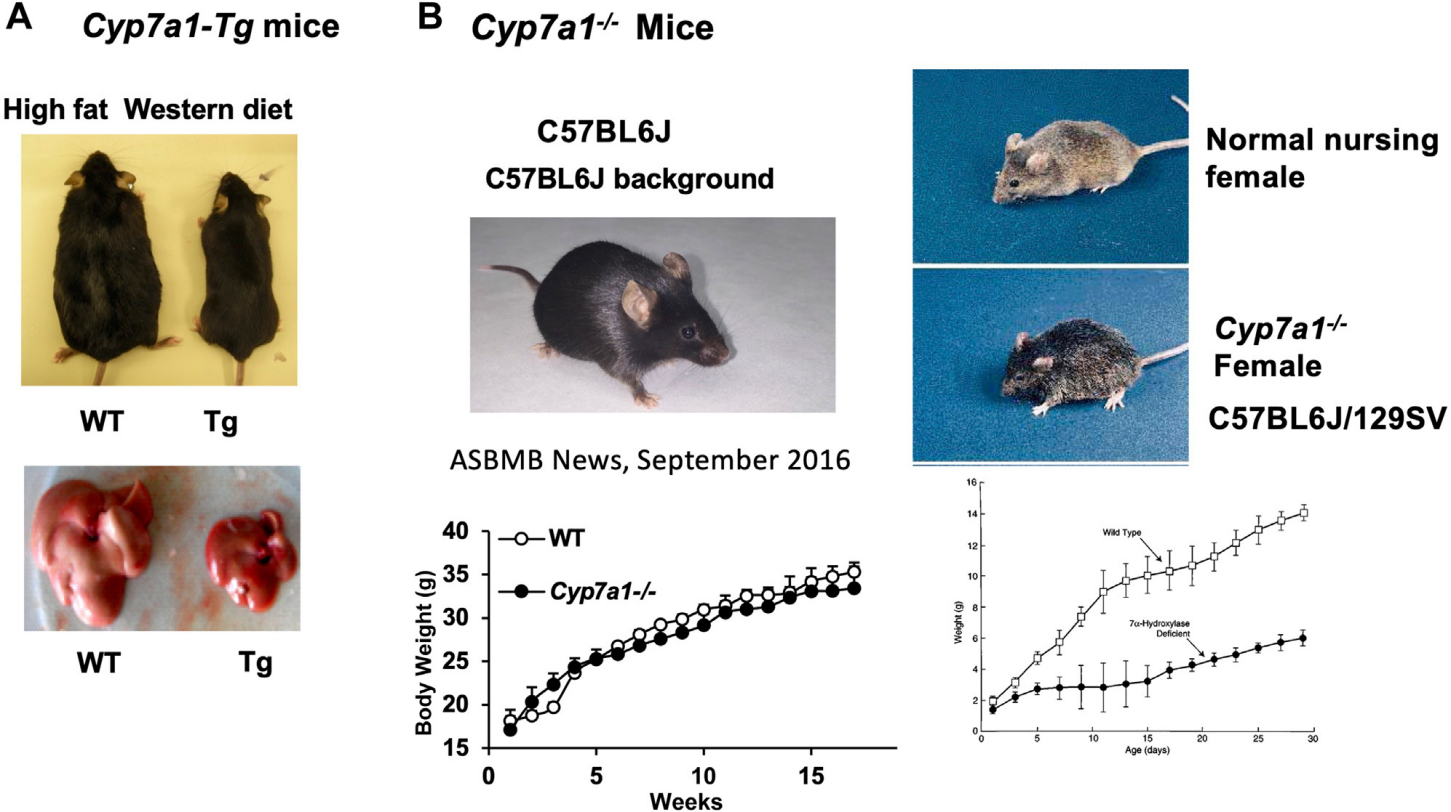
**Cyp7a1 genetic mouse models used in my research.***A*, Cyp7a1 transgenic mice are resistant to Western high diet–induced obesity and diabetes. *B*, Cyp7a1-deficient mice shown phenotype differences in C57BL/6J pure genetic background (*left*) *versus* the mixed background (*right*).

The role of bile acids in liver metabolism and diseases has been the subject of intense study in the last 20 years (36). We used mouse models to study the effects of fatty liver disease on bile acid pool size and composition. As a biochemist and molecular biologist, I did not have experience in breeding mice. I was fortunate to hire highly capable research assistants, Erica Owsley and Shannon Boehme, to breed mice and set up our mouse colonies in 2010. Transgenic mice overexpressing rat CYP7A1 cDNA had an increased bile acid pool size by 2.5-fold but reduced CA and increased bile acid hydrophobicity. To our surprise, these Cyp7a1 transgenic mice were protected from high-fat and high-cholesterol diet–induced obesity, hepatic steatosis, and insulin resistance (Fig. 4*A*) (42). Furthermore, increasing CDCA, a more potent FXR agonist compared with CA, may activate FXR to markedly induce intestinal FGF15 (24-fold), which stimulated adipose tissue browning in these animals.

We then studied the effects of reduced bile acid synthesis on metabolism using *Cyp7a1-*knockout mice to test if these animals were more prone to developing fatty liver and diabetes on a high-fat and high-cholesterol diet. The C57BL/6J background is commonly used to study metabolic disease in mice. We therefore backcrossed *Cyp7a1*-knockout mice in a mixed background (Cyp7a1^tm1Rus^/6J, B6/129Sv; Jackson Labs) to C57BL/6J mice for seven generations to achieve mice with about 99% pure C57BL/6J background (Fig. 4*B*) (43). To our surprise, our *Cyp7a1*-deficient mice survived well and did not have the malnutrition phenotypes and mortality rates as originally described by David Russell’s laboratory (44). These mice had a smaller bile acid pool size (60% of the wildtype mice) and upregulated expression of the genes involved in alternative bile acid synthesis, with a more hydrophilic bile acid composition in the gallbladder bile. Also, to our surprise, these mice had improved glucose sensitivity and reduced liver triglycerides and fecal bile acid excretion on a high-fat Western diet. This study suggests a critical role of bile acid composition, rather than just bile acid pool size, in maintaining metabolic homeostasis and protecting against high fat diet–induced metabolic disorder. Furthermore, our study showed that *Cyp7a1*-deficient mice accumulated free cholesterol, had more severe hepatic inflammation, oxidative stress, apoptosis, and increased fibrosis when fed a methionine–choline-deficient diet to induce nonalcoholic steatohepatitis fibrosis (45). Notably, adenovirus-mediated reconstitution of CYP7A1 in these mice reduced hepatic-free cholesterol and oxidative stress and reversed hepatic inflammation and fibrosis, suggesting CYP7A1 plays a key role in maintaining cholesterol homeostasis and protecting against hepatic inflammation and injury, and diet-induced obesity and fatty liver disease.

Mutations of the *CYP7B1* gene in neonates and infants cause severe cholestasis. Mutations of the C*YP7B1* gene were found in spastic paraplegia type 5 patients (46). However, the *Cyp7b1*-deficient mice are normal but accumulate 25-hydroxycholesterol and 27-hydroxycholesterol in the liver and serum (47). We thus bred *Cyp7a1-* and *Cyp7b1-*double knockout mice to test if these double knockout mice will have abnormal metabolic phenotypes or altered bile acid pool and bile acid synthesis. Preliminary data showed that these double knockout mice were phenotypically normal and had a smaller bile acid pool like *Cyp7a1*-single knockout mice. We thus speculate that other 7α-hydroxylases may exist to produce bile acids in the absence of Cyp7a1 and Cyp7b1.

### Studying gut microbiota in bile acid metabolism

In collaboration with Frank Gonzalez and Andrew Patterson (Pennsylvania State University), we studied the effects of the intestinal-specific FXR agonist, fexaramine, on bile acid synthesis and metabolism in mice. My postdoc, Preeti Pathak, found that fexaramine treatment markedly increased LCA, FGF15, FGF21, and GLP-1 secretion, improved insulin and glucose tolerance, and promoted white adipose tissue browning in wildtype mice (48). Analysis of the gut microbiota identified that fexaramine induces the LCA-producing bacteria *Acetatifactor* and *Bacteroides*. These bacteria have both 7α- and 7β-dehydroxylase activities, which convert CDCA and ursodeoxycholic acid (UDCA) to LCA. Fexaramine treatment effectively improved lipid profiles, improved insulin and glucose tolerance, and increased adipose tissue browning and energy metabolism in obese and diabetic mice, whereas antibiotic treatment completely reversed the beneficial metabolic effects of fexaramine and reduced taurolithocholic acid production. LCA is a p otent Takeda G protein–coupled receptor 5 (TGR5) (a bile acid–activated G protein–coupled receptor 1) agonist that stimulates GLP-1 secretion and improves metabolism. This study demonstrated that an intestine-specific FXR agonist reshaped the gut microbiota to activate TGR5–GLP-1 signaling to improve metabolism, and that the gut microbiota plays a critical role in bile acid metabolism to regulate metabolic homeostasis in health and disease.

### Discovery of FXR and TGR5 crosstalk

Little was known about the regulation of TGR5 expression. We found that FXR and TGR5 were coexpressed in enteroendocrine L cells. We hypothesized that FXR and TGR5 may crosstalk in the intestine to regulate bile acid synthesis and hepatic metabolism (49). In this *JBC* article, Preeti used the FXR–TGR5 dual agonist INT-767 to study bile acid synthesis and GLP-1 secretion in wildtype, *Fxr* knockout, and *Tgr5* knockout mice. We found that INT-767 effectively stimulated GLP-1 secretion more than that of the FXR agonist obeticholic acid or the TGR5 agonist INT-777. Interestingly, INT-767 reduced the expression of classic bile acid synthesis genes but induced genes of the alternative pathway to decrease TCA and increase tauromuricholic acid. Surprisingly, Preeti found that activation of FXR induced TGR5 expression in the ileum. We identified an FXR-responsive element in the mouse *Tgr5* gene promoter. This study uncovered a novel mechanism by which FXR activation induced TGR5 expression to stimulate GLP-1 secretion and improve hepatic glucose and lipid metabolism. Activation of both FXR and TGR5 may represent an effective therapy for managing hepatic steatosis, obesity, and diabetes. Shannon then generated *Fxr* and *Tgr5* double knockout mice to study bile acid synthesis and regulation. Surprisingly, these double knockout mice had reduced liver lipids but increased serum cholesterol (50). We found that both *Cyp7a1* and *Cyp8b1* mRNA levels were increased, and bile acid pool size was increased with increased TCA but decreased tauromuricholic acids. RNA sequencing revealed liver fibrosis and inflammation. Gene expression levels were increased compared with wildtype mice. These *Fxr*- and *Tgr5*-double knockout mice had impaired regulation of bile acid synthesis when fed cholestyramine and CA diets and may be a useful model to study liver fibrosis. These studies serve as a proof of concept that bile acid–based drugs may be developed for treating metabolic liver diseases (51). Drugs targeting FXR and TGR5 signaling are in clinical trials for nonalcoholic steatohepatitis fibrosis and nonalcoholic fatty liver diseases (36).

## International collaborations

Our success in cloning CYP7A1 cDNA attracted much attention from investigators all over the world, and I received hundreds of requests for CYP7A1 cDNA plasmids. At that time, PCR was yet to be invented, and cDNA plasmids were needed for Northern blot hybridization to detect and quantify mRNA expression levels in tissues. We distributed these plasmids to any investigator that requested them.

**Figure 5:**
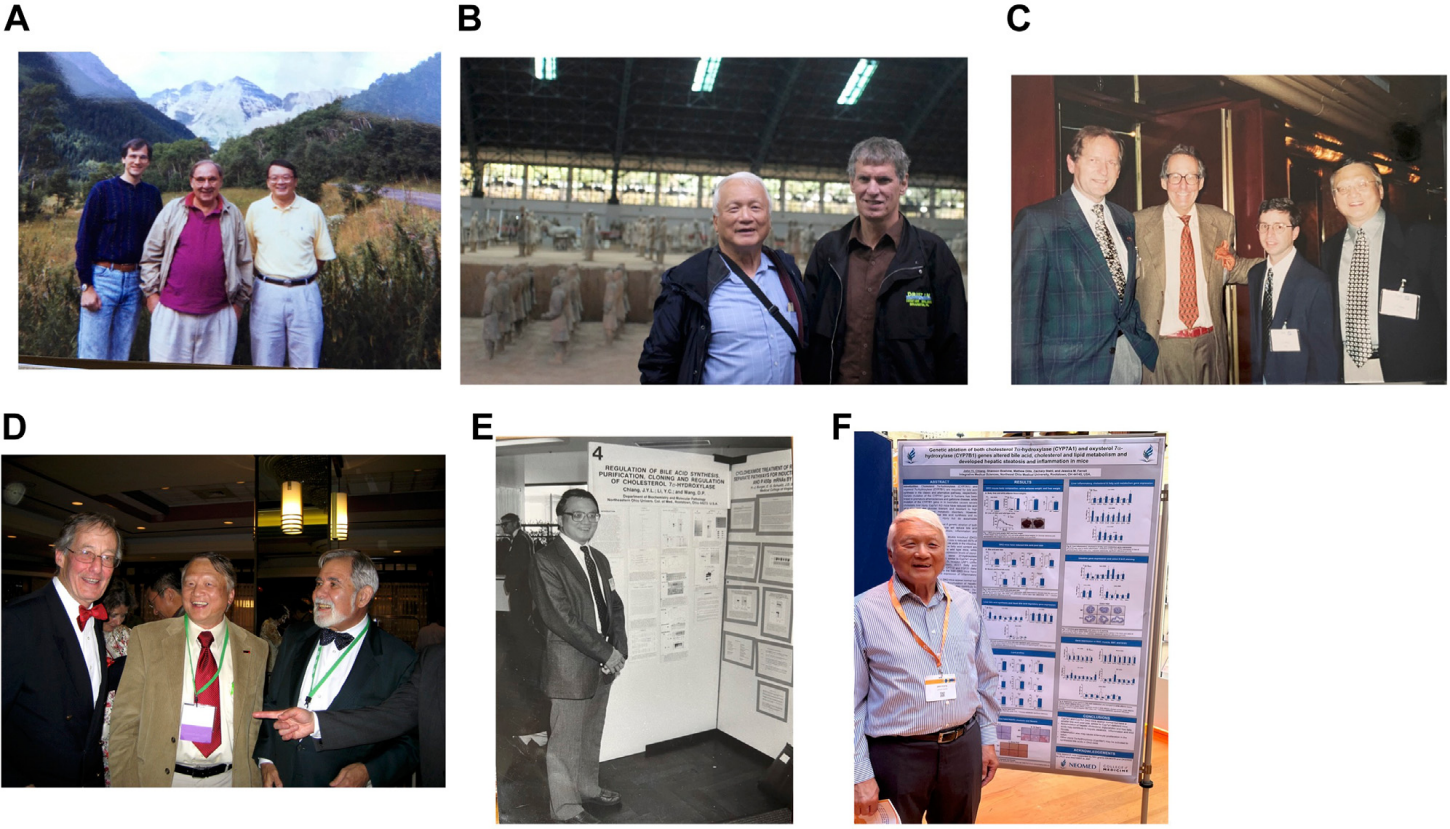
**Collaborators in bile acid research.***A*, the author with Mike Pandak and Reno Vlahcevic at Kern Lipid Conference in Aspen, 1992. *B*, the author with Frank Gonzalez in Xi’an, China, in 2017. *C*, the author with Curt Einarsson, Alan Hofmann, and Paul Dawson (*left to right*) at Falk bile acid meeting 2000 in The Hague, Netherlands. *D*, the author with Alan Hofmann and Gerry Salen in Digestive Disease Week meeting in Chicago, 2007. *E*, the authors’ first Falk bile acid meeting presentation in Freiburg, Germany, 1990. *F*, the author’s latest Falk bile acid meeting presentation in Amsterdam, Netherlands, 2022.

Professor Phil Hylemon was the first to call me and invited me to Richmond, VA to meet a group of gastroenterologists led by Professor Reno Vlahcevic, MD including gastrointestinal research fellow Michael Pandak, MD at Medical College of Virginia (Fig. 5*A*). We collaborated to study transcriptional regulation of bile acid synthesis for 10 years and published a total of nine articles together. They asked me to participate on a program project application to study molecular mechanisms of regulation of bile acid synthesis. I remembered that Phil’s house had no air conditioning, and we worked on the front patio under 95° heat. The program project was funded, but my subproject was dropped because of my location far from Richmond. My subproject was subsequently submitted and funded as my second R01 grant in 1994.

Professor Giovani Galli (University of Milan) sent his student Maurizio Crestani to my laboratory as a Fulbright Scholar in 1991 to 1992. Maurizio returned to my laboratory as a postdoc from 1993 to 1995 and joined my laboratory again in 1996 to 1997 as a research instructor. Maurizio published seven articles with me and made significant contributions to our study of transcriptional regulation of CYP7A1. Professors Ingemar Bjorkhem and Curt Einarsson (Karolinska Hospital) (Fig. 5*B*) sent a graduate student YiZeng Yang to my laboratory to work on CYP8B1 regulation. I was approached by Dr Simon Choi of the Chonnam University in South Korea to collaborate on the CYP7A1 projects, and we published 11 articles together. These internal collaborations were fruitful and exposed us to international bile acid research communities. Hoechst/Aventis invited me to Frankfurt to serve as a consultant for several years, and they provided research grants as well as eight patent applications of *CYP7A1* gene structure, sequence, and their uses. I was invited to many drug companies and universities to give seminars on cloning of Cyp7a1. I learned from these experiences, **L7: you should be generous in sharing your reagents and expertise to promote the work of others, which, in turn, will help your own research**.

### Falk Symposium bile acid meetings

Falk Symposium International Bile Acid Meetings were initially organized by Hans Popper, Herbert Falk, and other Germany researchers. These meetings have been the premier bile acid and digestive disease meeting since 1970. Dr Falk Pharma is a Freiburg-based drug company that specializes in bile acid drugs for liver diseases and has been the sponsor of these bile acid meetings. Over the years, I have attended 12 Falk Symposium International Bile Acid Meetings, mostly as an invited speaker. I remembered at the Falk Symposium in Freiburg in 1992, Frank Gonzalez and I joined the afternoon hiking trip and took a train ride to the Staufen train station in the Black Forest, Bavarian, where the marching band led us to the town center and the mayor gave a welcome speech. Then Dr Herbert Falk led us hiking in the Black Forest to Munstertal and had a German dinner, and there was folk music and dancing in a barn. These symposia opened my eyes to the world of bile acids and drug therapy for digestive diseases and the opportunity to meet many leading bile acid investigators in the world and had significant impact on my bile acid research. I first met Frank when I was a postdoc. We met frequently at many P450 and nuclear receptor meetings around the world. In 2017, we attended a meeting in Xi’an, China, and toured the old Chang An Wall and Terracotta Warriors Museum (Fig. 5*B*). I admire his significant contributions to P450 and nuclear receptor research and greatly appreciate his long friendship. We have collaborated and published five articles together.

I met Alan Hofmann (Univeristy of California San Diego), for the first time, at Basel airport on our ride to Freiburg for my first Falk Symposium meeting in 1990 and every Falk meeting I attended afterward (Fig. 5, *C* and *D*). Dr Hofmann was regarded as the encyclopedia of bile acids, was a great mentor and role model who inspired me to do bile acid research, and provided advice to me frequently. The field lost a giant when Dr Hofmann passed away in 2021. I learned the importance and molecular biology of bile acid transporters from Paul Dawson (now at Emory) (Fig. 5,*C*). Paul Dawson made significant contributions in the discovery and cloning of the ASBT and OSTα/β genes that code for bile acid transporters in the intestine. Phil, Paul, and I often spoke at the same bile acid and cholesterol conferences.

I meet Professors Ingemar Bjorkhem and Curt Einarsson (Karolinska Institutes) at my first Falk symposium and many Falk Symposia afterward. They are pioneers in bile acid and cholesterol metabolism and treatment of liver diseases. They invited me to Stockholm several times, including a presentation at the Nobel Conference on transcriptional control of lipid homeostasis sponsored by the Nobel Assembly in Huddinge, Sweden, 2003, with many distinguished investigators in lipid metabolism. We published three articles together.

Professor Gerry Salen (University of Medicine and Dentistry of New Jersey) (Fig. 5*D*) was a dedicated physician scientist who treated gallstone disease and cholestatic liver disease patients with UDCA and rare genetic cholesterol storage diseases, sitosterolemia (ABCG5/G8 mutations), and cerebrotendinous xanthomatosis (*CYP27A*1 gene mutations) patients with CDCA. Gerry told me he used to drive to Pennsylvania Amish country to deliver UDCA to his patients with gallstone disease and cholestasis. We collaborated and published four articles together. Dr Salen passed away in 2020. He was another mentor of mine and had a significant impact on my research in cholesterol and bile acids. I always remember his smile and his enthusiasm in bile acid and cholesterol research. Figure 5*E* shows my first Falk Symposium Bile Acid Meeting presentation in Freiburg, in 1990 and my latest Falk Symposium Bile Acid Meeting presentation in Amsterdam, 2022 (Fig. 5*F*).

## Networking

Being situated in a small medical school in rural Ohio, networking with other investigators was especially important for me. I learned from my mentors the importance of presenting at professional society meetings. I believe it is beneficial for a PhD scientist to join not only societies of basic science but also those focusing on clinical sciences. When I was an Assistant Professor, I joined the basic science societies American Society for Biochemistry and Molecular Biology and American Society for Pharmacology and Experimental Therapeutics (ASPET). Later, when my research began to address bile acids and liver diseases, I joined science societies that are more clinical in orientation: American Association for the Study of Liver Disease (AASLD), the American Gastroenterology Association, the American Diabetes Association, and the Endocrine Society. I brought every graduate student and postdoc to the annual Federation of American Societies of Experimental Biology/Experimental Biology and AASLD meetings to present their work and compete for travel and poster awards. I published most of our article in society journals, starting in the basic science journals: *JBC*, *JLR*, *Drug Metabolism and Disposition*, *Molecular Pharmacology*, *American Journal of Physiology*, and so on and later in more clinical journals: *Hepatology and Gastroenterology*. Through these associations, I was able to introduce my students and postdocs to leading investigators in the field.

In the early 1980s, Chinese researchers in North America organized the Society for Chinese Biological Scientists in America (SCBA) to promote networking and recruitment of graduate students and postdocs. This Society runs biannual international meetings in the United States, China, and Taiwan, and many Nobel Laureates and US National Academy of Sciences members are invited to give keynote speeches. The society also provides travel awards to graduate students and postdocs. I am a chater member of SCBA. Chinese liver researchers in the United States recently organized a Chinese American Liver Society, a division of SCBA, which holds annual research symposia to discuss liver research in person and *via* Zoom meetings. These professional societies gave me opportunities to network with many distinguished scientists in the liver research field.

**Figure 6:**
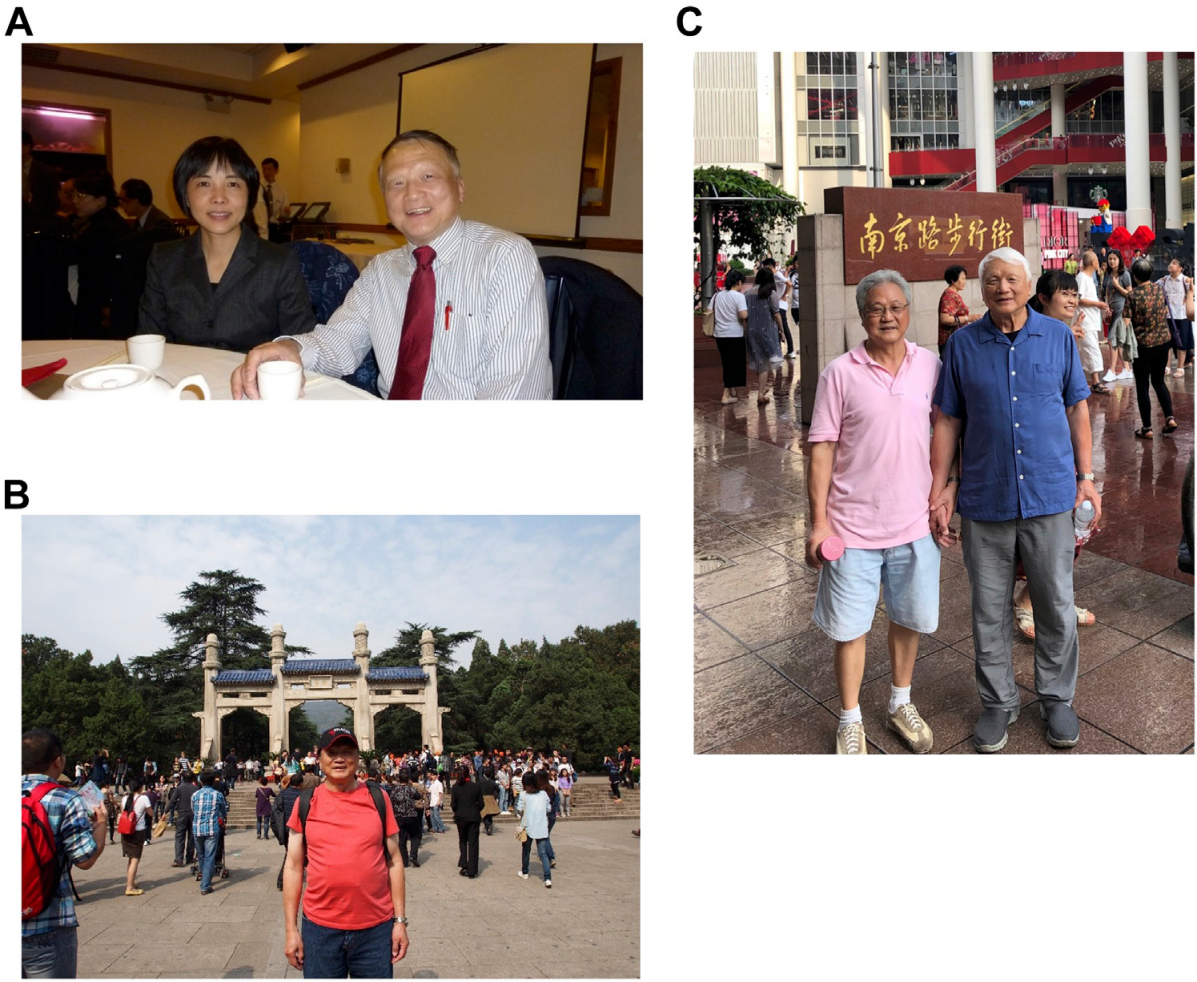
**Author visited to his birthplace in China.***A*, author with Huiping Zhou in DDW meeting in Chicago, 2009. *B*, author toured Nanjing, China, in 2012. *C*, author with his youngest brother in Shanghai, China in 2019.

I met Huiping Zhou who joined the liver center at Virginia Commonwealth University in 2001. We share common research interest in bile acid metabolism and became close friends (Fig. 6*A*). In 2012, Huiping organized a lecture tour in Nanjing, my birthplace and visited beautiful West Lake in Hangzhou, my father’s birthplace in China. It was the first time I returned to Nanjing, where I was born 65 years ago (Fig. 6*B*). In 2019, Huiping organized another lecture tour in Guangzhou and went on to attend the International SCBA Conference in Kunming, China, where my parents stayed for a year during the Sino-Japanese War. I visited my brother in Shanghai afterward (Fig. 6C). Wen Xie, chair of Pharmaceutical Sciences at the University of Pittsburgh School of Pharmacy, contacted me when he moved to Pittsburgh as Assistant Professor. We are very close, separated by 100 miles, and share common research interest in nuclear receptors in drug metabolism and detoxification.

I served on many NIH and American Heart Association and VA Merit award review study sections. I served as a standing member of the NIH Biochemistry Study Section (1996–2000) and a charter member of the Hepatobiliary Pathophysiology Study Section (2004–2008). Members of these study sections are distinguished investigators in their fields, many of them had served as the President of AASLD. Networking with these influential investigators had significant impact on my research career. Richard Hanson (Case Western Reserve University Medical Center), an Associate Editor of *JBC*, invited me to serve on the editorial board of *JBC*. Dick was a nice man and great biochemist and my mentor. I always remember his smile and wits. I have served on the editorial board of Hepatology for 17 years, several AASLD committees, and was secretary/treasurer of the Drug Metabolism Division of ASPET. I participated several times in AASLD Hill Day to meet with congressional members on Capitol Hill to discuss various issues about liver diseases, treatments, and managements, and to lobby for more NIH funding. I will continue serving on the editorial boards of Hepatology and Hepatology Communications and am an Executive Associate Editor of Liver Research. Another lesson **L8: serving on study sections and society journal editorial boards is our duty as scientists** **funded** **by taxpayers’ money**.

## Mentoring

I attribute my success in teaching and research to the good mentoring I had received. I was particularly grateful to the mentorship of Dr Hutterer in both teaching and research, which had a significant impact on my academic career. I received every award available to NEOMED faculty: the Faculty Research Award in 2001, the Liebelt/Wheeler Award for Faculty Excellence in 2013, and the Lifetime Achievement Award from the NEOMED Alumni Association in 2017. In 2013, I was appointed the very first Distinguished University Professor by the Board of Trustees of NEOMED.

My mentoring of students, postdocs, and the younger generation of investigators has made tangible contributions to the biomedical research community. The principal investigator has the important job of mentoring graduate students and postdocs in their early careers, rather than simply pushing them to work in the laboratory. The principal investigator needs to be patient to teach them how to solve problems, generate hypotheses, analyze data, interpret results, write articles, and apply for grants. All my students and postdocs who were eligible to apply for fellowships were successful in receiving National Research Service Award or American Heart Association postdoc fellowships. In weekly laboratory meetings, I first gave an update on current events in our research area, then discussed laboratory management issues and problems, and scheduled oral presentations and discussions of research data and journal articles. The Department of Biochemistry and Molecular Biology was combined with Microbiology and Immunology, then combined again with Physiology to form the Department of Integrative Medical Sciences (IMS) in 2006. I helped to recruit two junior faculty with research expertise in bile acids and nuclear receptors to IMS in 2008. I organized a focused liver research group on nuclear receptors in metabolic diseases. We had regular meetings of faculty, graduate students, and other research staff to discuss research data and journal articles. Unfortunately, the liver research group did not expand in the IMS department, which focused more on cardiovascular physiology. Although this liver research group remains small with only four tenure-track faculty, its investigators have been highly successful in NIH grant funding and liver research. The lesson **L9: being in a small pond has the disadvantages of slow growth and competition for limited resources**.

**Figure 7:**
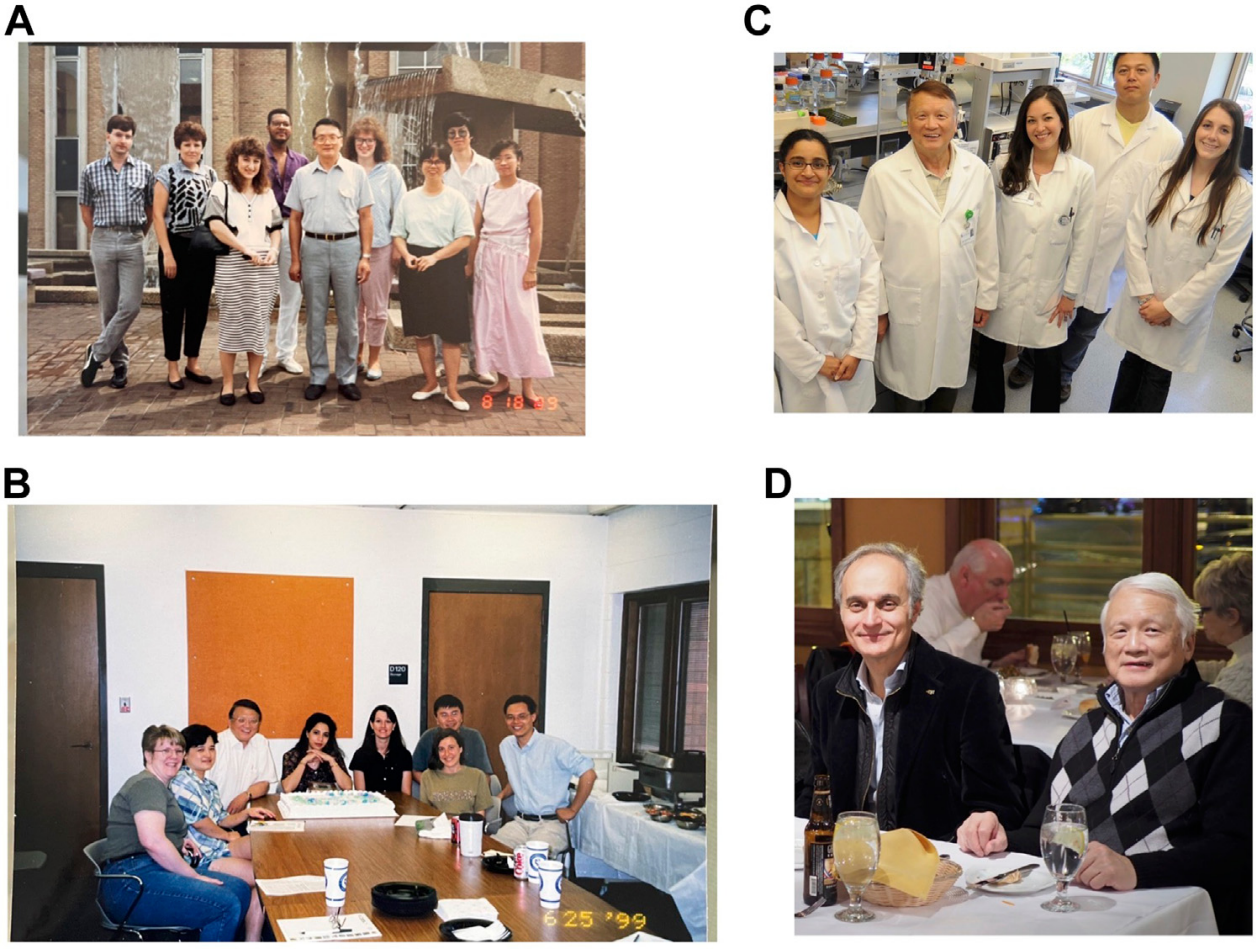
**Author’s lab members over the years.***A*, author’s first group of students and staffs in 1989: Yun Chun Li (PhD 1991) is the first on the *right*, Dan Ping Wang (PhD 1996), the third from the *right*, and research staffs, Rich Vernell, the first on the *left* and Wynne Miller, the third from the *left*. *B*, author’s group in 1999. *Left* to *right*, Ronda Kimmel (technician) and Wenling Chen (PhD 2002), author, Azeta Sadeghpour (PhD 1998), Diane Stroup (Assistant Professor 1998), Ming Zhang (PhD 2002), Maria Marrapodi (postdoc 1999), Zhang Liang Wu (PhD 2000). *C*, my long-time laboratory members, 2011 to 2022, *Left*: *left to right*, Preeti Pathak (PhD 2013 and postdoc 2013–2018), author, Shannon Boehme (research assistant and laboratory manager 2010–present), Tiangang Li (PhD 2006 and postdoc 2006–2011), and Jessica Ferrell (postdoc 2011–2015, Assistant Professor 2015–present), taken in 2001. *D*, author with Maurizio Crestani (University of Milan), taken in 2017.

NEOUCOM jointly formed a graduate program in biomedical sciences with Kent State University. All graduate students were enrolled through Kent State University. The faculty from NEOUCOM and Kent State University taught graduate classes at both campuses and had joint seminars and journal club courses rotating between the two campuses, which are separated by about a 15 min drive. I trained 12 PhD students, 3 master’s degree students, and 20 postdocs (Fig. 7). Several graduate students and postdocs of mine went on to have their own successful academia careers and supervise their own independent laboratories, among which Yan Chun Li (University of Chicago), Maurizio Crestani (University of Milan), and Tiangang Li (University of Oklahoma) are good examples.

I met Grace Guo (Rutgers University) when she was a postdoc in Frank Gonzalez’s laboratory. She joined the University of Kansas Medical Center as an Assistant Professor. We collaborated on bile acid metabolism using genetic knockout mouse models for many years. Dr Curt Klaassen, then Chair of the Department of Pharmacology, Toxicology, and Therapeutics at the University of Kansas Medical Center asked me to serve on the External Advisory Board for their NIH-funded Center of Biomedical Research Excellence (COBRE) Program. I visited Kansas City every year during the three phases of the COBRE award and followed the progress of many COBRE trainees, including Wen-Xing Ding, Grace Guo, Xiao-Chao Ma, Tiangang Li, Lisa Zhang, and so on, from assistant professor to associate professor, and some to full professor. They all were successful in obtaining R01 grants after finishing their COBRE training. I wrote letters supporting their promotion and tenure applications and critiqued their grant applications. I actively nominated my students and many junior investigators for society awards and wrote supporting letters for their grant applications and recommendation letters for their job applications and permanent residency applications. I appreciated their trust and friendship and am happy for their successes in academic research.

## Completing my 4 decades of dedication for bile acid research

It has been an amazing journey in bile acid research. In August 2022, I decided to retire, but I had a plan for continuing bile acid research at NEOMED. I did not want to end my research journey by closing my laboratory; instead, I turned over my laboratory—including reagents, staff, genetically modified mice, and an active NIH R01 grant to Jessica Ferrell, who has worked with me since 2011, first as a postdoc and as a tenure-track assistant professor at NEOMED in 2022. Jessica is now well versed in bile acid and metabolism research.

**Figure 8:**
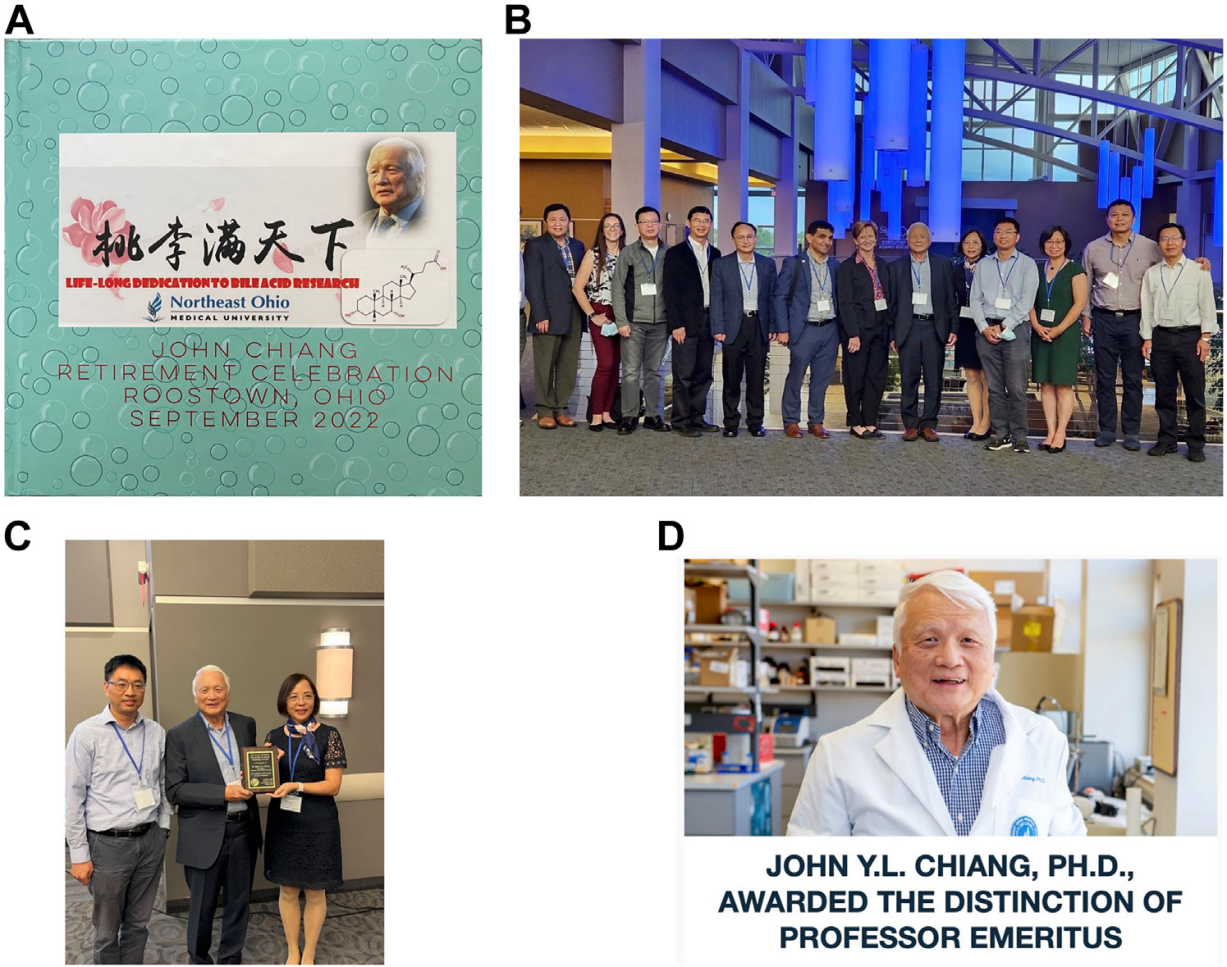
**The symposium organized by liver research friends celebrating the author’s retirement at NEOMED, September 24, 2022, Rootstown, OH.***A*, the Chinese characters read: “Peaches and plums all over the world” meaning “Mentoring students all over the world”. *B*, group picture with speakers, *left* to *right*: Ping Zhang (NEOMED), Jessica Ferrell (NEOMED), Yan Chun Li (University of Chicago), Wen Xie (University of Pittsburgh), Yanqiao Zhang (NEOMED), Takhar Kasumov (NEOMED), keynote speaker Laura Nagy (Cleveland Clinic), me, Huiping Zhou (Virginia Commonwealth University Medical School), Wen-Xing Ding (University of Kansas Medical Center), Grace Guo (Rutgers University), Tiangang Li (University of Oklahoma), and Xiaochao Ma (University of Pittsburgh), 2022, NEOMED. *C*, Chinese American Liver Society President Wen-Xing Ding and past president Huiping Zhou presented a plaque of Lifetime Achievement in Liver Research during the Symposium. *D*, the Board of Trustee of NEOMED awarded the distinction of Professor Emeritus to the author.

On September 24, 2022, to my surprise and delight, Wen Xie, along with a group of research colleagues and friends, organized a symposium to celebrate my retirement (Fig. 8, *A*–*C*) in Rootstown. My liver research friends, former students, and NEOMED colleagues. We had about 50 people who attended this symposium. I was honored that Laura Nagy (Cleveland Clinic) came to give the keynote speech. Laura and I first met at a NIH study section meeting over 20 years ago, and since then we often ran into each other at the Cleveland airport while traveling to study section meetings and conferences. Several other NEOMED colleagues, postdocs, and graduate students also presented their research. We had a great day of science, fun, good food, and endless great memories. I thank them whole-heartedly for their warm friendships. Another surprise, my daughter-in-law, Cindy, organized a “Tribute,” which collected video recordings of well wishes from over 20 friends, colleagues, and my family members. This concludes my 52 years of academic life in the United States with 44 years dedicated to bile acid research. My lesson **L10: friendships are for life, and you will relish that forever**.

## Key reflections on my long and winding journey in bile acid research

My journey in research was not always a smooth ride. I encountered many roadblocks. First, I had difficulty purifying CYP7A1 and assaying for enzyme activity in the beginning. I had to develop a more sensitive activity assay method and change animal models in order to purify and clone Cyp7a1. Initially, I had difficulty obtaining an NIH grant, but I never gave up and finally my perseverance paid off and my grant application was funded on the third try. Looking back, I think my grant writing skill was not good at that time, and I did not have sufficient support and mentoring on grant writing at a small medical school. My application for promotion to Associate Professor and tenure was denied in 1982. I had to appeal to reverse the committee’s decision to be promoted to Associate Professor with tenure in 1983. I had difficult cloning CYP7A1 cDNA. I took a detour with a sabbatical leave to learn molecular biology and cloning techniques and then successfully cloned CYP7A1 cDNA quickly afterward. I adopted many molecular biology techniques new to the bile acid field to study the transcriptional regulation of CYP7A1. I then took another detour in using human primary hepatocytes to study bile acid metabolism and discovered many surprising differences between the regulation of rat and human CYP7A1. Later, I took another detour to develop mouse models for defining bile acid signaling in liver disease. We discovered many additional surprises from our knockout mouse studies. These experiences taught me **L11: there are many obstacles where only commitment, patience, and creativity will allow you to overcome them.**

Altogether, I have published a total of 190 articles, which have been cited 18,880 times (h-index 73, Google Scholar). My thematic review of bile acids for *JLR*, “Bile acids: regulation of synthesis” has been cited over 1580 times (52). I am satisfied with my research productivity considering I had a small group and was supported by a small research budget. I attribute my lifelong academic success to hard work, perseverance, networking, and the good mentoring that I received. I only applied for two NIH RO1 grants; both grants were funded and renewed continuously for 23 and 32 years, respectively. In 2010, I was awarded a 10-year MERIT Award (R37) from the National Institute of Diabetes and Digestive and Kidney Diseases, in recognition of continuous high productivity and creativity in research projects. In recognition of my contribution to excellent teaching and research, NEOMED appointed me the first Distinguished University Professor in 2013. Throughout my career, people asked me why I spent my entire academic life at NEOMED, and why I did not move to a larger and more prestigious research I nstitution. My answer was because NEOMED provided me with an environment that I felt comfortable with to do my research without interruption. I did have an opportunity to move to a famous medical school 20 years ago but decided to stay at NEOMED so my research would not be interrupted by moving**. My final lesson L12: being a big fish in a small pond may have some advantages over being a small fish in a bigger pond.**

## Final thought

I thought this was an amazingly long journey for a 23-year-old young Chinese man who came to the United States to pursue his American dream and succeeded beyond his imagination. The Board of Trustees of NEOMED awarded the distinction of Professor Emeritus to me (Fig. 8*D*). I can now relax and enjoy my retirement with my wife, two sons, and five grandkids, living close by in the city of Westlake on the West Shore of Lake Erie, Ohio. I am delighted to be selected as the recipient of the 2023 E. Leong Way Emeritus Travel Award, a Scientific Achievement Award of ASPET that recognizes my lifelong dedication to research, to attend the 2023 ASPET meeting in St Louis, MO. This award will provide me an opportunity to interact with ASPET attendees. I will also continue to read and review articles for my society journals.

## Conflict of interest

The author declares no conflicts of interest with the contents of this article.
